# Subalpine training at 1,200 m altitude for 8 weeks: impact on aerobic and anaerobic capacity, cardiac function, and hemodynamics in male paralympic biathletes

**DOI:** 10.3389/fphys.2026.1719907

**Published:** 2026-07-15

**Authors:** Runji Wang, Qianlan Zhang, Ye Liu

**Affiliations:** 1 School of Physical Education and Health, Changchun Normal University, Changchun, Jilin, China; 2 Scientific Research Department, Changchun Normal University, Changchun, Jilin, China

**Keywords:** subalpine training, para-biathlon, aerobic and anaerobic capacity, cardiac function, hemodynamics

## Abstract

Subalpine training plays an important role in the physical conditioning and performance enhancement of athletes. Previous studies have analyzed the characteristics of changes in cardiac and electroencephalographic indices, but there is a gap in current research to investigate the changes in energy metabolism, cardiac function, and hemodynamics in biathletes with disabilities who underwent 8 weeks of subalpine training at 1,200 m altitude. It is important to explore the changes of these indicators to guide the scientific training and competition of disabled biathletes. To investigate the effects of 8 weeks of subalpine training at 1,200 m altitude on aerobic and anaerobic capacity, cardiac function, and hemodynamics of male Paralympic biathletes, and to make scientific recommendations for the training and participation of para-biathletes. Energy metabolism, cardiac function, and hemodynamics are crucial physiological indicators reflecting athletes’ physical stress, exercise capacity, and circulatory regulation. Therefore, these indicators were measured to reveal the physiological adaptation of athletes during high-intensity exercise. 14 male biathletes [age (23.9 ± 2.4), height (176.3 ± 7.6) cm, and body mass (58.2 ± 8.9) kg] from the national winter Paralympic training team were trained for 8 weeks at a subalpine altitude of 1,200 m. Their aerobic and anaerobic capacity, cardiac function, and hemodynamic parameters were assessed before, during, and after the subalpine training, and their data were compared using repeated measures analysis. After two weeks of subalpine training, athletes had significantly elevated cardiac index (p < 0.001), variability in output per beat (p < 0.001), and thoracic fluid content (p < 0.001), and significantly decreased output per beat (p < 0.001) and left ventricular ejection time (p < 0.001). Additionally, we found that after 8 weeks of subalpine training, their cardiac index was significantly higher than before subalpine training (p < 0.001). There was no significant change in beat-to-beat output (p = 0.904), beat-to-beat output variability (p = 0.866) or thoracic fluid content from (p = 0.208) before subalpine, and the left ventricular ejection time was significantly lower than before subalpine (p = 0.003). After eight weeks of subalpine training at 1,200 m, the male Paralympic biathletes demonstrated a significant decrease in aerobic capacity indices, including peak oxygen uptake (VO_2_peak) and ventilation (p<0.05), while no significant change was observed in anaerobic capacity indices such as peak power, mean power and fatigue index (p>0.05). The study findings suggest that subalpine training for two weeks can lead to reduced circulatory system volume and inflammatory pleural effusion in Paralympic biathletes. However, 8 weeks of subalpine training at 1,200 m altitude can enhance stress resistance, adaptability, cardiac function, and cardiac reserve while significantly reducing aerobic capacity in Paralympic biathletes. When conducting subalpine training, the physical function monitoring of Paralympic biathletes might be available to focus on cardiovascular function and thermodynamics-related microcirculatory indicators. Additionally, a comprehensive assessment of the circulatory and respiratory systems’ functional status could be performed to maintain and improve training quality and competitive status.

## Introduction

The snow sports competition for the 2026 Winter Paralympic Games was held in Italy at the Antolc-Anterselva Olympic venue, which has an average altitude of 1,200 m to 1,600 m. The analysis of the subalpine environment at an altitude of about 1,200 m to 1,600 m is of great value to the paralympic biathletes in their preparation for the 2026 Winter Paralympic Games. It will have a profound impact on the development of China’s snow physical fitness program. Accordingly, the altitude of approximately 1,200 m in this study is consistent with the “low elevation” range defined by the current academic community (Evgeny et al., 2019; Burtscher et al., 2018). For subalpine training, it is generally divided into pre-subalpine plateau training (Before entering the subalpine environment training), subalpine plateau training (Subalpine training in progress), and post-subalpine plateau training (After leaving the subalpine environment).

Several studies have demonstrated that a decrease in the partial pressure of oxygen is associated with reduced maximum aerobic capacity and muscular power in humans, which is particularly pronounced during the early stages of exposure to 1200 m subalpine altitudes before acclimatization occurs (Mazzeo, 2008). In addition, 1200 m subalpine altitudes are associated with an increased risk of illness, persistent fatigue, weight loss, and overtraining (Mazzeo, 2008; Czuba et al., 2014). These effects are of particular relevance to Winter Olympic sports, with biathlon and cross-country skiing being the disciplines that are most directly impacted by changes in altitude (Chapman et al., 2010). It has been observed that many athletes who reside at 1,200 m subalpine altitudes for three or more weeks undergo various physiological adaptations that improve their ability to cope with the stresses associated with altitude, such as energy supply and cardiac function, thus enabling them to engage in more intense training to enhance their athletic performance (Flaherty et al., 2016; Foss et al., 2017). Despite the fact that altitude training can significantly enhance aerobic work capacity, it may also lead to a reduction in athletic performance or have an impact on competitive ability due to athletes’ maladaptation to altitude training, elevated sympathetic excitability under hypoxic stimulation, increased changes in humoral regulatory hormones, and increased blood viscosity (Bădău et al., 2016; Chapman et al., 2010). In 1200 m subalpine altitudes environments, athletes can obtain adequate intensity stimulation from two sources: the hypoxic effects on physiological functions and the ability to maintain training intensity levels similar to those of low-altitude regions, thereby avoiding problems such as physiological disorders and decreased athletic performance that are often seen in traditional plateau training. Previous studies have reported that training at an altitude of 1200 m exerts positive effects on biathletes with regard to physical fitness and physiological function (Evgeny et al., 2019; Bădău et al., 2016; Lunghi et al., 2019).

Athletes with physical disabilities in subalpine environments may experience uneven blood distribution and inadequate muscle blood supply throughout the body (Toresdahl et al., 2019). Athletes with physical disabilities, particularly those with lower-limb amputation (unilateral or bilateral, complete amputation) and spinal cord injury, may exhibit impaired sympathetic vascular regulation and altered peripheral blood distribution under subalpine hypoxic conditions. Due to reduced skeletal muscle mass, impaired vasomotor control, and altered venous return, these athletes may experience uneven systemic blood distribution and insufficient muscle perfusion during exercise compared with able-bodied athletes. These physiological characteristics have been demonstrated in previous cardiovascular screening and hemodynamic studies of Paralympic athletes, using invasive and noninvasive methods including cardiac output monitoring, Doppler ultrasound, and near-infrared spectroscopy (Sawczuk et al., 2023). Hypoxic stimulation, to a certain degree, can lead to the inhibition of sympathetic portions of the vagal nervous system, decreased venous blood pressure, increased vascular tension, and disruption of neuromuscular conduction and muscle trophic processes. Although subalpine training has been gradually introduced into the training regimens of disabled athletes, and subalpine training has been reported to be closely related to changes in physiological functional status and exercise capacity of disabled athletes, there have been few studies conducted on the effects of subalpine training at 1,200 m altitude on the aerobic and anaerobic capacity, cardiac function, and hemodynamics of Paralympic winter athletes in the snow physical fitness categories (Toresdahl et al., 2019; Clarsen et al., 2022).

To address this gap in the literature, the present study aims to investigate the effects of an 8-week subalpine training program at 1,200 m altitude on the aerobic and anaerobic capacity, cardiac function, and hemodynamics of male Paralympic biathletes to provide specific training recommendations for the preparation of Paralympic biathlon events based on trends observed in relevant indicators.

## Methods

### Participants

The study included a total of 14 athletic athletes (mean ± SD age, 23.97 ± 2.34 years) from the China national disabled cross-country skiing and biathlon training team, all of whom had met the entry requirements for biathlon entry points for the Beijing 2022 Winter Paralympics before and after participating in the study. Among them, there were 8 athletes in a seated position (with physical disabilities in seated LW12) and 6 athletes in a standing position (with physical disabilities in standing LW8) (**Table 1**). The study was approved by the Ethics Committee of the Capital University of Physical Education and Sports, and all participants provided written informed consent after a thorough explanation of the nature and objectives of the study. Institutional ethical approval was also given in accordance with the 1964 Declaration of Helsinki.

**Table 1:** Basic information of research subjects.

Serial Number	Age/Years old	Height/cm	Weight/kg	Years of participation in sports	Location of injury	Disability classification
1	22	183	65	2	Amputation of the right lower leg	LW12
2	22	175	58	2	Amputation of the right lower leg	LW12
3	27	170	55	4	Amputation of the right thigh	LW12
4	26	178	55	3	Amputation of the right thigh	LW12
5	26	175	43	3	Amputation of both thighs	LW12
6	20	178	65	4	Atrophy of the right thigh	LW12
7	30	182	67	5	Left lower leg amputation	LW12
8	25	185	77	5	Left thigh amputation	LW12
9	19	170	55	2.5	Right hand amputation	LW8
10	18	172	50	2	Right hand amputation	LW8
11	24	178	62	2.5	Right hand amputation	LW8
12	20	171	58	3	Right hand amputation	LW8
13	23	170	58	2.5	Right hand amputation	LW8
14	21	170	55	2.5	Right hand amputation	LW8
Mean value	24.07 ± 3.74	174.07 ± 5.12	54.86 ± 8.21	3.07 ± 1.13		

### Training protocol

The study was conducted between January 15 and March 15 in 2019, for a total of 8 weeks, with 18 male athletes training and undergoing testing at the Bayi Snow Park in Mudanjiang City, Heilongjiang Province, over a period of 8 weeks. The base of the Baigao Ski Resort in Mudanjiang City, Heilongjiang Province, has an altitude of 1,200 meters.

The exact altitude of each testing site was recorded using a calibrated GPS-enabled altimeter. The fraction of inspired oxygen (FIO_2_) was assumed to be 20.93% (consistent with ambient atmospheric oxygen concentration). The inspired oxygen partial pressure (PIO_2_) was estimated as:q

PIO_2_ = (Barometric Pressure − Water Vapor Pressure) × FIO_2_

Standard barometric pressure at sea level is 760 mmHg; PIO_2_ decreases by approximately 7 mmHg per 1,000 m ascent.

Accordingly, the estimated partial pressure of inspired oxygen (PIO_2_) at Bayi Snow Park, Mudanjiang, was approximately 62.3 mmHg.

pre-subalpine plateau training (Before entering the subalpine environment training), subalpine plateau training (Subalpine training in progress), and post-subalpine plateau training (After leaving the subalpine environment). A total of five tests were conducted during the study, with the first test (M1) being conducted during the pre-subalpine plateau phase, followed by tests at weeks 2 (M2), 4 (M3), 6 (M4), and 8 (M5) of training. The weekly training plan was adjusted based on daily weather conditions and included low to moderate intensity aerobic training focused on adaptation, specific speed and strength training aimed at increasing intensity, high-intensity transition session simulation training focused on improving specific performance, and relaxation training aimed at energy recovery. The content and load of training A and training B are different in the 3rd-4th week, that is, the pre-competition stage (**Table 2**).

**Table 2:** Stage load arrangement of 8-week subalpine training.

Phase	Time	Training objectives	Training content		Load capacity		Load intensity
			A	B	A	B	
Pre-competition training	Week 1-2	Adaptation	Aerobic training	Aerobic training	Ski 286.5 km; Shoot 180 min Shooting under load 120 min	Ski 200 km; Shoot 180 min Shooting under load 60 min	Medium and low
	Week 3-4	Intensity	Specialized speed and strength	Strength training	Ski 243 km; Shoot 320 min Shooting under load 160 min	Ski 200 km; Shoot 240 min Shooting under load 240 min	Medium and high
In-game	Week 5–6	Performance	Match	Match	Ski 226 km; Shoot 180 min	Ski 206 km; Shoot 180 min	High
Post-game adjustment	Week 7-8	Recover	Aerobic recovery training	Aerobic recovery training	Ski 243 km; Shoot 420 min	Ski 243 km; Shoot 420 min	Medium and low

Based on previous experience in high-altitude training and the training goals during the preparation period, the 8-week sub-high-altitude training is divided into 5 small cycle sections. Each small cycle section consists of 2 training stages, and a reduction in training load is made in the second training stage. The training content includes cross-country skiing training, specialized shooting training, strength training, and functional training. The training load intensity is classified as low intensity training (LIT), moderate intensity training (MIT), and high intensity training (HIT).

The training objectives and contents of the short-term training blocks are presented in **Table 3**: 1) The accumulation stage of aerobic endurance (M1 - M3). The training objective at this stage is to facilitate sub - high - altitude aerobic adaptation and refine low - intensity techniques. Snow training primarily comprises moderate - to - low - intensity aerobic gliding (40% - 60% of the maximum heart rate, HRmax), supplemented by specialized intermittent endurance training. Special shooting training mainly employs shooting training based on the resting heart rate (RHR), concentrating on precision practice in a stable state. Dry fire training (DF), which is simulated shooting training without ammunition when the weapon is unloaded, is frequently utilized to familiarize athletes with weapon operation and practice the shooting action process. Preload shooting training (PLST), with a load intensity controlled at 60% - 100% of HRmax, focuses on the shooting ability of athletes under high-intensity loads. Strength training (ST) involves training the upper - body strength, core strength, and lower - body strength of athletes at 80% - 85% of the one - repetition maximum (1RM). Functional training (FT) aims to enhance the efficiency of key movements, specialized adaptability, and injury resistance of athletes in the combined event of cross-country skiing and rifle shooting. Through training that mimics specific action patterns of the event, it reinforces the training system for body coordination, stability, and energy transfer. 2) The transformation and reduction adjustment stage of specialized speed endurance (M4 - M5). The training objective at this stage is to cultivate the athlete’s specialized sprint ability and optimize the efficiency of the gliding - shooting transition. The intensity of snow training reaches over 95% of the maximum speed, emphasizing the ability to sustain speed in the later stage. Special shooting training maintains the content and load arrangement of the previous stage. Strength training is conducted at 60% 1RM based on the specific characteristics of the sport, focusing on strength endurance practice while reducing the load.

**Table 3:** Arrangement of training load and content in sub-plateau training.

Time	Week 1		Week 2		Week 4		Week 6		Week 8	
	Morning	Afternoon	Morning	Afternoon	Morning	Afternoon	Morning	Afternoon	Morning	Afternoon
Monday	LIT 25 km	LIT 10 km	LIT 25 km	PLST 1 h	LIT 25 km	PLST 1 h	LIT 15 km	LIT 15 km	LIT 25 km	DF 1 h
Tuesday	LIT 25 km	ST	LIT 25 km	ST	LIT 25 km	ST	FT	ST	LIT 25 km	ST
Wednesday	LIT 25km	DF1h	LIT 40km	DF 1h	LIT 40km	PLST 1h	LIT 25km	DF 1h	LIT 40km	DF 1h
Thursday	MIT 8 km	LIT 7 km	HIT 6.5 km	LIT 15 km	MIT 6.5 km	DF 1 h	MIT 8 km	LIT 10 km	LIT 6.5 km	DF 0.5 h
Friday	FT	LIT 15 km	FT	LIT 15 km	FT	DF 1h	LIT 20 km	Testing match	FT	DF 0.5 h
Saturday	LIT 20 km	DF 1 h	LIT 25 km	DF 1 h	LIT 25 km	Rest	LIT 20 km	DF 0.5 h	LIT 25 km	DF 0.5 h
Sunday	Rest	Rest	Rest	Rest	Rest	Rest	Rest	Rest	Rest	Rest
Sliding distance	135km		151.5km		121.5km		113km		121.5km	
Shooting duration	2h		3h		4h		1.5h		3.5h	

The training load schedule and content for weeks 3, 5, and 7 are the same as those for weeks 4, 6, and 8.

### Aerobic capacity test

To quantify the aerobic capacity of standing and sitting para-biathlon athletes with physical disabilities at different stages of altitude exposure, standardized aerobic capacity tests were performed at five key time points (M1–M5), with peak oxygen uptake (VO_2_peak, unit: mL·kg^−1^·min^−1^) as the core evaluation index and ventilatory threshold (VT, unit: mL·kg^−1^·min^−1^) as an auxiliary evaluation index to comprehensively reflect the athletes’ aerobic metabolism level. Standing athletes performed tests on a ski simulator in a standing position, while sitting athletes used a seated ski simulator. The test procedure followed standard exercise physiology guidelines, as detailed below:

### Test equipment and calibration

A calibrated portable metabolic analysis system (Model: K5, Manufacturer: COSMED S.r.l., Italy) was used to real-time monitor and record ventilation and metabolic indicators, including VO_2_, minute ventilation (VE), respiratory exchange ratio (RER), and respiratory rate (RR). An electronic heart rate (HR) monitor (Model: Polar Vantage V3, Manufacturer: Polar Electro Oy, Finland) was synchronized to record HR at 1 Hz. One hour before the test, the metabolic system was calibrated for gas (using standard O_2_ and CO_2_ gases) and flow to ensure data accuracy; the HR monitor was closely attached to the subject’s skin and calibrated before use.

Additionally, a ski simulator (Model: Para Ski Simulator S1, Manufacturer: Hebei Shenghua Jian Sports Technology Co., Ltd., China) was pre-adjusted for precise speed control—standing athletes used the simulator in a standing position and sitting athletes in a seated position. A lactate analyzer (Model: YSI 1500 SPORT, Manufacturer: YSI Inc., USA), disposable blood collection needles, and tubes were prepared for immediate blood lactate determination, with the analyzer calibrated to ensure a detection error within ±0.1 mmol/L.

### Pre-test preparation

Subjects were instructed to avoid strenuous exercise, alcohol, smoking, and high-sugar/high-fat foods 24 hours before the test, and to ensure adequate sleep (≥8 h). One hour before the test, they consumed a light, easy-to-digest meal (e.g., bread, milk) to avoid fasting or overeating and supplemented 500–800 mL of warm water to maintain hydration.

Ten minutes before the test, subjects entered the test environment (temperature: 20–22°C, humidity: 50%–60%, consistent with the altitude test site), wore the metabolic mask and HR monitor, and performed a warm-up: 5 min of walking at 3.0 km/h, followed by 3 min at 4.0 km/h, and joint mobilization/stretching to prevent injury. After warm-up, subjects rested for 3 min to stabilize HR and respiration, with resting HR, VO_2_, and RER recorded as baseline data.

### Test protocol

An incremental load protocol was adopted on the ski simulator: initial speed 4.0 km/h (0° slope equivalent), increased by 1.0 km/h every 3 min until exhaustion. Standing athletes performed in a standing position, while sitting athletes in a seated position. Exhaustion was defined as inability to maintain speed, obvious dyspnea, lower limb fatigue, RER ≥1.15, and HR ≥95% of predicted maximum heart rate for ≥30 s.

During the test, the metabolic system recorded VO_2_, VE, RER, and RR every 1 min (average value); HR was recorded every 30 s. Thirty seconds before the end of each load stage, 20 μL of fingertip blood was collected to measure blood lactate concentration. A dedicated observer monitored subjects for discomfort (e.g., dizziness, nausea, chest tightness), with immediate test termination if symptoms occurred.

### Post-test recovery and data collation

After the test, subjects performed a 10-min cool-down walk at 2.5–3.0 km/h followed by full-body stretching to relieve fatigue, with HR and respiration monitored until returning to resting levels ±10 beats/min.

VO_2_peak was defined as the highest VO_2_ value recorded (average of 30 consecutive seconds). Abnormal data (e.g., equipment failure, test interruption) were excluded, with retesting arranged after ≥24 h. VE, RER, HR, and blood lactate data at each time point were collated for combined analysis with VO_2_peak to comprehensively evaluate aerobic capacity.

### Anaerobic capacity test

Anaerobic capacity of standing and sitting para-biathlon athletes with physical disabilities was assessed using the Wingate Anaerobic Test, synchronized with the five time points (M1–M5) of aerobic capacity testing to ensure data consistency. Considering the athletes’ physical conditions, different test equipment and protocols were adopted: standing athletes (with intact lower limbs) used a Wingate anaerobic power bicycle, while sitting athletes (with lower limb disabilities but intact upper limbs) used a hand-crank anaerobic ergometer. The specific protocols are as follows:

### Equipment and calibration

Different classic equipment was selected based on the athletes’ physical conditions to ensure test feasibility and accuracy: 1. For standing athletes (intact lower limbs): A calibrated Wingate anaerobic power bicycle (Model: Monark 894E, Manufacturer: Monark Exercise AB, Sweden) was used, which is a classic and widely used equipment in anaerobic capacity testing. The bicycle was adjusted to a standing position with adjustable handrails to ensure the athletes’ stability during riding.

2. For sitting athletes (lower limb disabilities, intact upper limbs): A calibrated hand-crank anaerobic ergometer (Model: Monark 881E, Manufacturer: Monark Exercise AB, Sweden) was adopted, a classic hand-operated equipment suitable for athletes with lower limb impairments. The ergometer was adjusted to a comfortable sitting height with a stable backrest and adjustable hand crank distance to adapt to the athletes’ upper limb movement range.

Both pieces of equipment were calibrated before each test according to the manufacturer’s guidelines to ensure accurate power measurement.

### Pre-test preparation

The pre-test preparation was consistent with the aerobic capacity test (avoiding strenuous exercise, adequate sleep, and light meals) to ensure the consistency of test conditions. After the recovery of the aerobic test, athletes rested for 30 min to fully restore their physical status. A targeted 5-min warm-up was performed based on the test equipment:

1. Standing athletes: They pedaled the Wingate anaerobic power bicycle at 60 rpm with no resistance, followed by 2–3 short maximal pedaling efforts (3–5 s) to activate anaerobic metabolism and warm up the lower limb muscles. 2. Sitting athletes: They rotated the hand crank of the anaerobic ergometer at 50–60 rpm with minimal resistance, followed by 2–3 short maximal cranking efforts (3–5 s) to activate anaerobic metabolism and warm up the upper limb muscles.

Warm-up was stopped immediately if the athletes felt any discomfort to avoid injury.

### Test protocol

The test was a 30-s maximal effort sprint for both groups, with protocols adjusted according to the equipment: 1. Standing athletes (Wingate anaerobic power bicycle): Resistance was set as 7.5% of the athlete’s body mass (a standard load for the Wingate test). Athletes were instructed to reach the maximal pedaling frequency within 3 s and maintain maximal effort for the entire 30 s. Real-time power output was recorded every 1 s by the bicycle. 2. Sitting athletes (hand-crank anaerobic ergometer): Resistance was set as 5.0% of the athlete’s body mass (adjusted to match the upper limb strength of athletes with lower limb disabilities). Athletes were instructed to reach the maximal cranking frequency within 3 s and maintain maximal effort for the entire 30 s. Real-time power output was recorded every 1 s by the ergometer.

A dedicated observer monitored the athletes throughout the test; the test was terminated immediately if the athletes showed obvious discomfort (e.g., dizziness, fatigue, or pain) to ensure safety.

### Key indicators and data collation

The same core anaerobic indicators were recorded for both groups to ensure the comparability of test results, including: peak anaerobic power (Ppeak, W·kg^−1^), mean anaerobic power (Pmean, W·kg^−1^), and fatigue index (FI, %). - Ppeak: The highest 1-s power output during the 30-s test; - Pmean: The average power output over the entire 30-s test; - FI: Calculated as [(Ppeak − minimum power in the last 5 s)/Ppeak] × 100%, reflecting the rate of anaerobic fatigue. Abnormal data (e.g., equipment failure, test interruption due to discomfort) were excluded, and retesting was arranged after ≥24 h to ensure data reliability.

### NICOM cardiac output test

**Figure 1:**
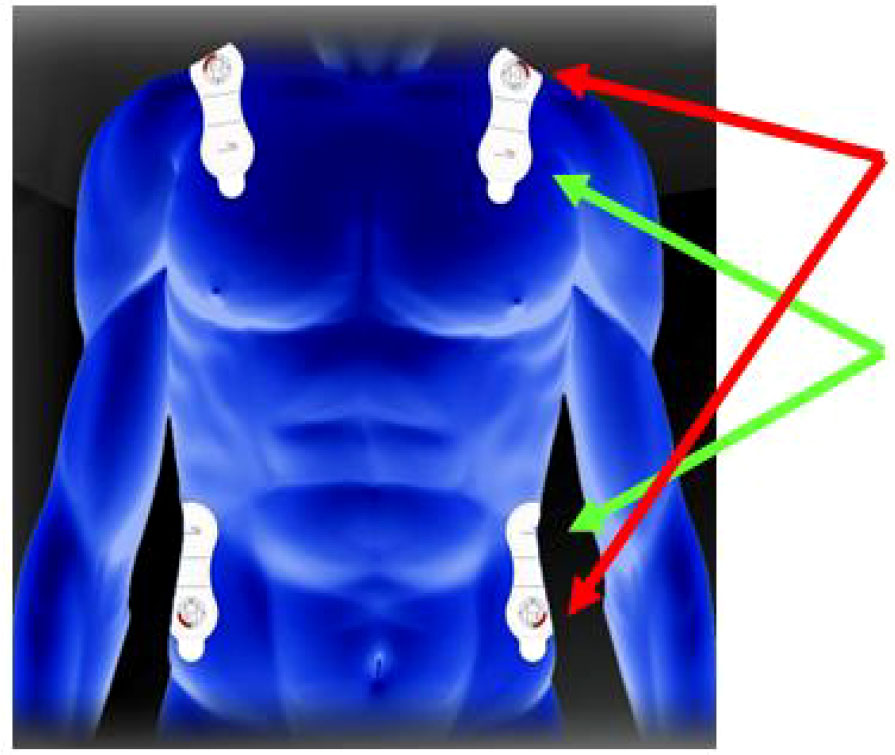
NICOM cardiac output test.

Noninvasive cardiac output monitoring (NICOM) has been validated against the thermodilution gold standard, with good correlation and acceptable agreement reported in previous studies (Squara et al., 2007; Binks et al., 2008; Cheung et al., 2015). Cardiac output testing was performed on all athletes using the NICOM Reliant non-invasive cardiac output testing system. In recent 10 years, the bioelectrical resistance method of non-invasive cardiac output monitor (NICOM) has gradually become more widely utilized for hemodynamic monitoring. The test required the application of four electrodes on the anterior side of the torso (around the heart), a high-frequency current at 75 kHz to the outer electrode of the human chest, and a simultaneous recording of the change in frequency by the inner electrode of the chest. Electrical and blood flow signals are converted to each other, allowing for the collection of continuous hemodynamic information, including cardiac output. The placement for the NICOM cardiac output test is shown in **Figure 1**.

The noninvasive cardiac output monitoring (NICOM) system provided the following key hemodynamic parameters:

Cardiac index (CI) represents the cardiac output per unit body surface area. The normal range is 3.0 to 3.5 liters per (minute·square meter).

The stroke volume index (SVI) reflects the pumping capacity of the heart per beat. The normal range is (60–80 ml).

Stroke volume variation (SVV), which reflects the changes in stroke volume caused by variations in intrathoracic pressure that affect the return blood volume, falls within the normal range (less than 10%).

Thoracic fluid content (TFC), which reflects the volume of fluid in the thoracic cavity, falls within the normal range of (5 to 15 ml).

**Figure 2:**
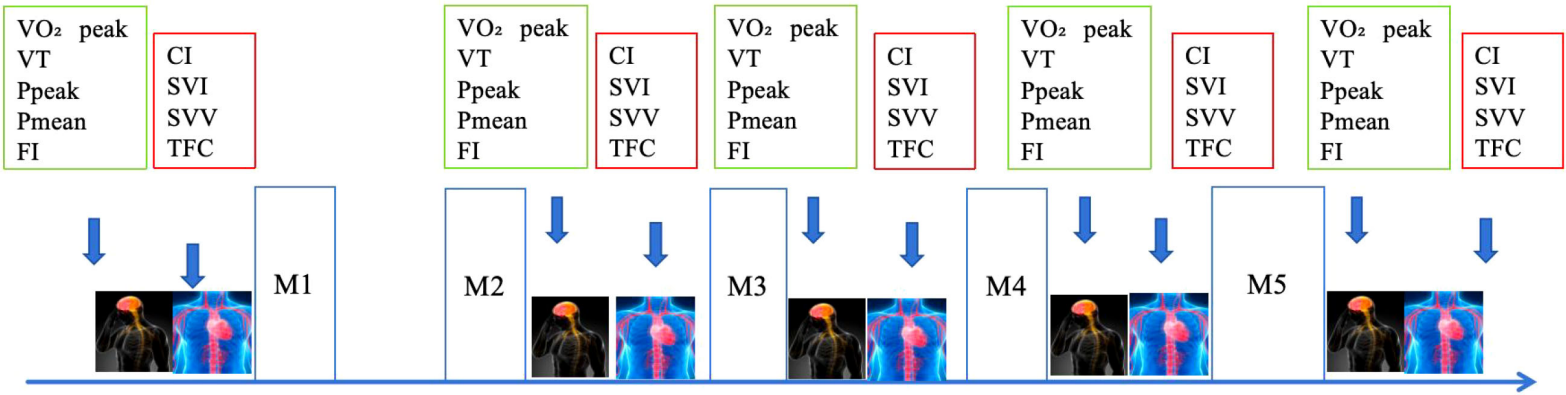
Test flowchart.

Following the aerobic, anaerobic capacity, athletes were required to maintain a seated position on a fixed seat. According to the requirements of the test system and combined with previous research methods, the skin of the designated electrode attachment sites was cleaned by a trained individual to ensure that the skin was free of hair and kept clean, and NICOM electrode patches were attached to the athletes. Four lead wires were then connected to the athletes’ bodies, and medical tape was used to secure the lead wires to prevent excessive shaking or falling off. Before starting the test, the system was allowed to run and perform automatic calibration while the athlete was verbally prompted to avoid sudden movements or speaking. Once the test started, the results were manually recorded, and the test was stopped when the system displayed the results normally. Three tests were conducted each time, and the average of the three tests was taken as the final test result. The testing process is shown in **Figure 2**.

### Statistical analysis

Data were entered into Microsoft Excel 2010 and analyzed with SPSS 26.0. Data were analyzed using parametric tests following confirmation of a normal distribution via the Shapiro-Wilks-W-test and are presented as mean ± standard deviation (SD). Data that met the normal distribution were further compared among the athletes using repeated-measures ANOVA. The results are presented as means with 95% confidence intervals (95% CI), and the statistical significance level was set at *p* ≤ 0.05.

## Results

The indicators presented in this section were obtained using the corresponding measurement methods. Cardiac index, stroke volume index, stroke volume variation, and thoracic fluid content were measured using the NICOM cardiac output test.

### Aerobic capacity

Statistical analysis of VO_2_peak and VT values across five time points was performed using one-way repeated measures analysis of variance (ANOVA) followed by Bonferroni *post-hoc* test. Differences in VO_2_peak and VT between time points were considered statistically significant at p <0.05 (**Table 4**). Results showed that both VO_2_peak and VT varied significantly among the five time points (VO_2_peak: F = 18.62, p<0.001; VT: F = 16.35, p<0.001). Compared with M1 (VO_2_peak: 60.0 ± 3.2 mL·kg^−1^·min^−1^; VT: 38.5 ± 2.1 mL·kg^−1^·min^−1^), VO_2_peak at M2 (58.2 ± 2.9 mL·kg^−1^·min^−1^) and VT at M2 (37.2 ± 2.0 mL·kg^−1^·min^−1^) showed a slight decrease but no statistical significance (p>0.05); VO_2_peak at M3 (55.8 ± 2.7 mL·kg^−1^·min^−1^) and M4 (54.0 ± 2.5 mL·kg^−1^·min^−1^), as well as VT at M3 (35.1 ± 1.8 mL·kg^−1^·min^−1^) and M4 (33.8 ± 1.7 mL·kg^−1^·min^−1^), were significantly lower than those at M1 (p<0.05 and p<0.01, respectively); VO_2_peak at M5 (57.0 ± 2.8 mL·kg^−1^·min^−1^) and VT at M5 (36.5 ± 1.9 mL·kg^−1^·min^−1^) were higher than those at M4 but still lower than those at M1, with no statistical significance (p>0.05). These results indicated that aerobic capacity (indicated by VO_2_peak and VT) gradually decreased with prolonged altitude exposure and partially recovered after descent from altitude.

**Table 4:** Changes in peak oxygen uptake (VO_2_peak) at different time points (M1–M5) during altitude exposure and post-altitude recovery.

Test time point	VO_2_peak (mL·kg^−1^·min^−1^, mean ± SD)	VT (mL·kg^−1^·min^−1^, mean ± SD)	Rate of change vs. M1 (%)	Statistical significance (p-value)
M1	60.0 ± 3.2	38.5 ± 2.1	—	—
M2	58.2 ± 2.9	37.2 ± 2.0	-3.0	p>0.05
M3	55.8 ± 2.7*	35.1 ± 1.8*	-7.0	p<0.05
M4	54.0 ± 2.5**	33.8 ± 1.7**	-10.0	p<0.01
M5	57.0 ± 2.8	36.5 ± 1.9	-5.0	p>0.05

Data are presented as Mean ± SD. VO_2_peak was expressed in mL·kg^−1^·min^−1^. *p<0.05 vs. M1; **p<0.01 vs. M1.

### Anaerobic capacity

Statistical analysis of anaerobic indicators (Ppeak, Pmean, FI) was consistent with that of aerobic capacity (one-way repeated measures analysis of variance (ANOVA) followed by Bonferroni *post-hoc* test, with statistical significance set at p<0.05) (**Table 5**). Results showed that Ppeak and Pmean of both standing and sitting athletes gradually decreased with prolonged altitude exposure (consistent with the changing trend of VO_2_peak), while FI gradually increased, indicating enhanced anaerobic fatigue. Specifically, for standing athletes, Ppeak decreased from 10.2 ± 1.1 W·kg^−1^ (M1) to 8.8 ± 0.8 W·kg^−1^ (M4) (a 13.7% reduction), and Pmean decreased from 6.8 ± 0.7 W·kg^−1^ (M1) to 5.7 ± 0.5 W·kg^−1^ (M4) (a 16.2% reduction); FI increased from 32.5 ± 3.1% (M1) to 39.5 ± 3.6% (M4) (a 21.5% increase). For sitting athletes, Ppeak decreased from 8.5 ± 0.9 W·kg^−1^ (M1) to 7.0 ± 0.6 W·kg^−1^ (M4) (a 17.6% reduction), and Pmean decreased from 5.6 ± 0.6 W·kg^−1^ (M1) to 4.6 ± 0.4 W·kg^−1^ (M4) (a 17.9% reduction); FI increased from 35.2 ± 3.3% (M1) to 42.8 ± 3.9% (M4) (a 21.6% increase). After descending from altitude (M5), Ppeak and Pmean of both groups showed partial recovery, and FI decreased, but did not return to pre-altitude (M1) levels, with no statistical significance compared with M1 (p>0.05). These findings indicated that hypoxic exposure during altitude training not only impairs aerobic capacity but also reduces anaerobic power and increases anaerobic fatigue, which can be partially reversed after returning to low altitude.

**Table 5:** Changes in anaerobic capacity indicators (Wingate Test) at different time points (M1–M5) in standing and sitting para-biathlon athletes.

Test time point	Standing athletes (wingate bicycle)	Sitting athletes (hand-crank ergometer)	Statistical significance (p-value)
	Ppeak (W·kg^−1^, Mean ± SD)	Ppeak (W·kg^−1^, Mean ± SD)	(vs. M1)
M1	10.2 ± 1.1	8.5 ± 0.9	—
M2	9.8 ± 1.0	8.1 ± 0.8	p>0.05
M3	7.5 ± 0.7*	7.5 ± 0.7*	p<0.05
M4	8.8 ± 0.8**	7.0 ± 0.6**	p<0.01
M5	9.6 ± 0.9	7.9 ± 0.8	p>0.05
	Pmean (W·kg^−1^, Mean ± SD)	Pmean (W·kg^−1^, Mean ± SD)	
M1	6.8 ± 0.7	5.6 ± 0.6	—
M2	6.5 ± 0.6	5.3 ± 0.5	p>0.05
M3	6.0 ± 0.5*	4.9 ± 0.4*	p<0.05
M4	5.7 ± 0.5**	4.6 ± 0.4**	p<0.01
M5	6.3 ± 0.6	5.2 ± 0.5	p>0.05
	FI (%, Mean ± SD)	FI (%, Mean ± SD)	
M1	32.5 ± 3.1	35.2 ± 3.3	—
M2	34.8 ± 3.2	37.5 ± 3.5	p>0.05
M3	37.2 ± 3.4*	40.1 ± 3.7*	p<0.05
M4	39.5 ± 3.6**	42.8 ± 3.9**	p<0.01
M5	35.6 ± 3.3	38.4 ± 3.6	p>0.05

Data are presented as Mean ± SD. Ppeak, Peak anaerobic power; Pmean, Mean anaerobic power; FI, Fatigue index. Standing athletes were tested with a Monark 894E Wingate anaerobic power bicycle and sitting athletes with a Monark 881E hand-crank anaerobic ergometer. *p<0.05 vs. M1; **p<0.01 vs. M1.

### Cardiac index

**Figure 3:**
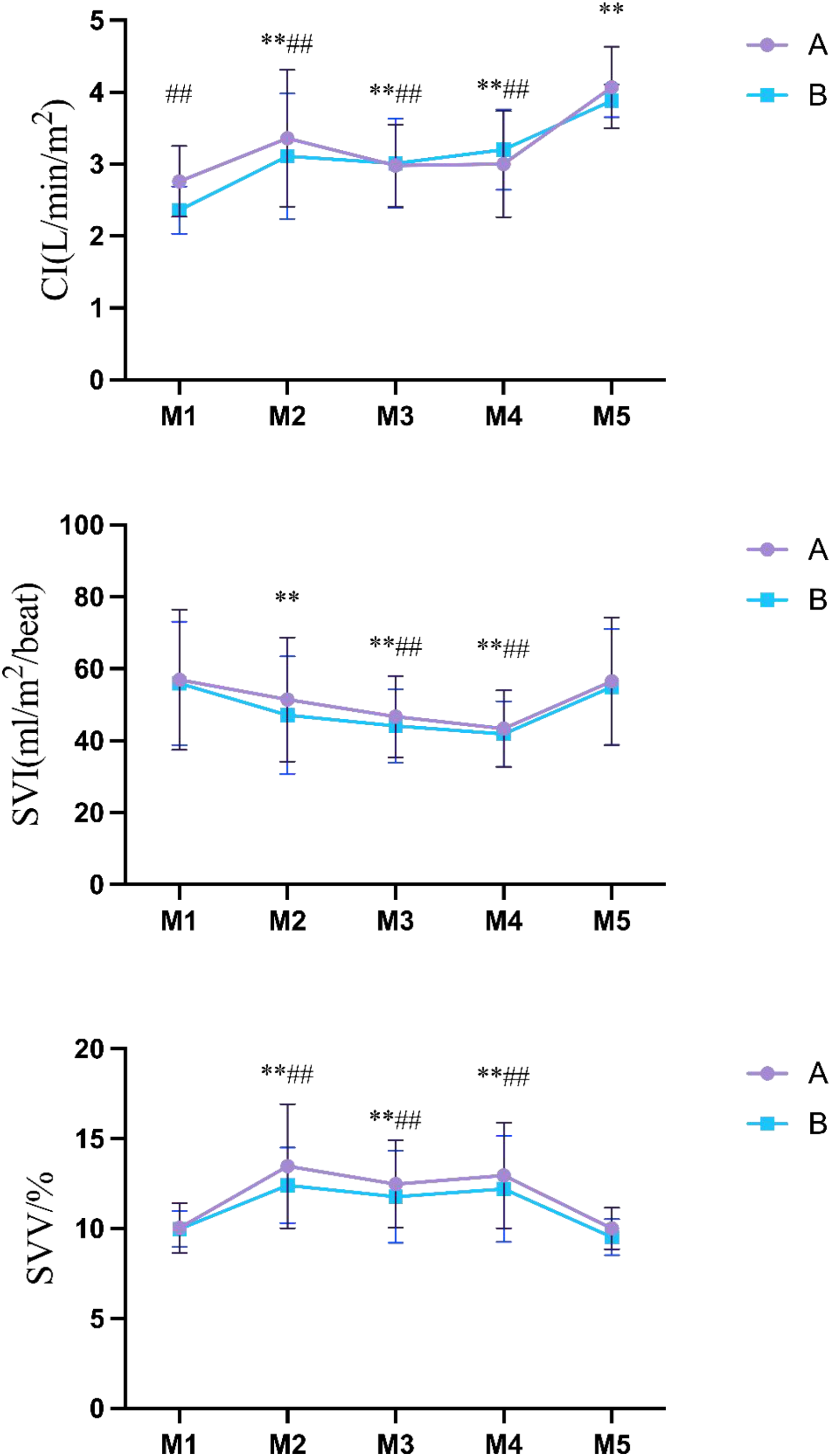
The 8-week subalpine training cardiac index (CI/SVI/SVV)-time trends of the examined Paralympic male biathletes. Significantly different compared to M1, **, p < 0.01. ##, p < 0.01, significantly different compared to M5.

CIs were significantly different across different time periods [F (1, 17) = 13.737, P < 0.001, η2 partial = 0.932]; whereby the CIs were significantly higher for M2, M3, M4, and M5 compared to M1 (P = 0.003, P < 0.001, P = 0.003, P < 0.001), while the CIs were significantly lower for M1, M2, M3, and M4 compared to M5 (P <0.001, P = 0.003, P<0.001, P<0.001) (**Figure 3**).

### Stroke volume index

SVI significantly differed at different time periods [F (1, 17) = 3.884, P = 0.03, η2 partial = 0.575]. We found that the SVI of M2, M3, and M4 was significantly lower than that of M1 (P < 0.001, P = 0.021, P = 0.037), and the SVI of M1 was not significantly different from that of M5 (P = 0.904). In addition, the SVI of M5 was significantly higher than that of M3 and M4 (P = 0.049, P = 0.029), and the SVI of M1 and M2 was not significantly different from M5 (P = 0.904, P = 0.112) (**Figure 3**).

### Stroke volume variation

Significant differences were observed in SVV at different time periods [f (1,17) = 10.047, P < 0.01, η2 partial = 0.649]. The SVV of M2, M3, and M4 was significantly higher than that of M1 (P < 0.001, P = 0.002, P = 0.001), and the SVV of M2, M3, and M4 was significantly higher than that of M5 (P < 0.001, P = 0.001, P = 0.001). No significant difference was found in SVV between M1 and M5 (P = 0.866) (**Figure 3**). There are only intra-group differences between A and B, but there should be no inter-group differences. Both ordinary and improved training contents are effective.

### Thoracic fluid content

**Figure 4:**
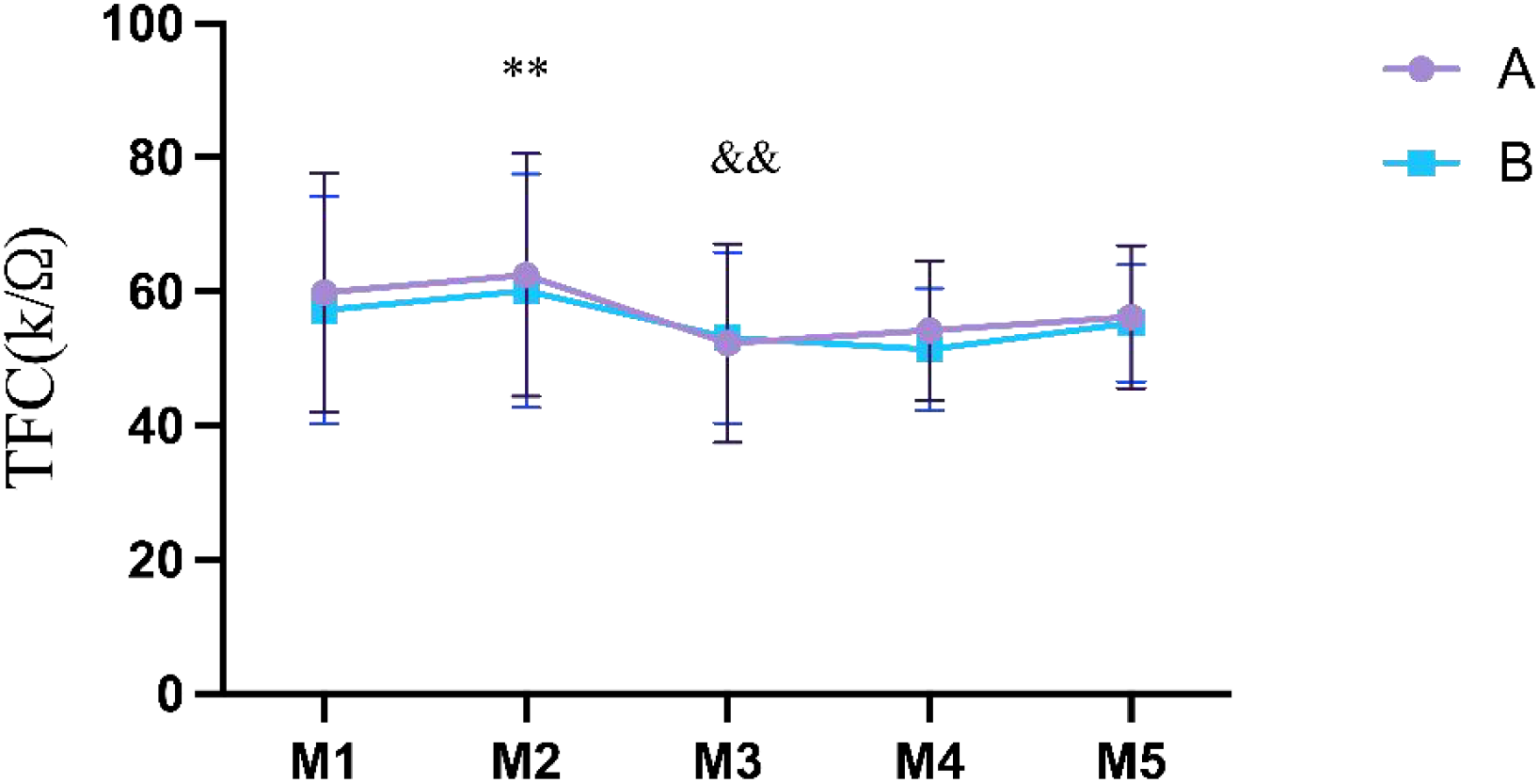
The 8-week subalpine training thoracic fluid content (TFC)-time trends in Paralympic male biathletes. && p < 0.01, significantly different compared to M2. Significantly different compared to M1, **, p < 0.01 , significantly different compared to M5.

The TFC was found to differ significantly at different times [F (1, 17) = 9.855, P < 0.01, η2 partial = 0.738]. The TFC of M2 was significantly different from M1 (P < 0.001), while the TFC of M3, M4, and M5 was not significantly different from M1 (P = 0.072, P = 0.263, P = 0.208). Additionally, we observed that the TFC of M3 was significantly lower than that of M2 (P = 0.020) (**Figure 4**).

## Discussion

### Aerobic and anaerobic capacity

This study investigated the changes in aerobic and anaerobic capacity of para-biathlon athletes during altitude exposure and post-altitude recovery, and the results are consistent with the physiological adaptation law of hypoxic exposure. The progressive decrease in VO_2_peak and VT with prolonged altitude stay (M1 to M4) and partial recovery after descent (M5) aligns with the findings of (Schuler et al., 2007; Burtscher et al., 2018; Bassett and Howley, 2000), who emphasized that a hypoxic environment reduces oxygen partial pressure, impairs oxygen transport and utilization, thereby decreasing aerobic capacity. The 10.0% reduction in VO_2_peak at M4 further confirms that long-term hypoxic stress significantly suppresses maximal oxygen uptake, which is the core indicator of aerobic capacity. Regarding anaerobic capacity, the gradual decrease in Ppeak and Pmean and increase in FI are consistent with (Sandbakk et al., 2013, 2011; Zhao et al., 2023), indicating that hypoxic exposure not only impairs aerobic metabolism but also reduces anaerobic power and accelerates fatigue. The more obvious reduction in sitting athletes’ Ppeak (17.6%) than standing athletes (13.7%) may be related to relatively weaker upper limb muscle mass and oxygen supply. The partial recovery of aerobic and anaerobic indicators at M5 is supported by (Lewis et al., 2015; Czuba et al., 2014; Yu et al., 2023), which suggests that the hypoxic-induced functional impairment is reversible when returning to a normoxic environment. However, the failure to restore to pre-altitude levels indicates that complete recovery requires longer time. This study supplements data for para-athletes’ altitude training, suggesting that altitude training should be individualized to avoid excessive hypoxic stress. Limitations include small sample size, and future studies should expand samples and monitor long-term recovery trends to provide more targeted guidance for para-athletes’ training.

### Cardiac index

The results of cardiac index tests showed that the CI of athletes after two weeks of subalpine training was significantly increased compared to before subalpine training, which might be related to the athletes’ central nervous system being in a hypoxic state and the sympathetic nerves being stimulated and excited due to the effect of the low-oxygen and low-pressure environment when they first reached the subalpine training, causing an increase in heart rate and cardiac output, which in turn compensated for the decrease in partial pressure of blood oxygen and maintained the oxygen supply (Mazzeo, 2008; Furian et al., 2022; Sharma et al., 2023). Studies have identified that various factors might cause the increase in heart rate at the first plateau, and the main cause of the increase in heart rate when athletes are at rest could be the stimulation of β-adrenergic receptor production in the heart through cardiac sympathetic nerves and adrenaline, which has a dominant role in the regulation of heart rate in athletes in subalpine training (Mazzeo, 2008; Christou et al., 2022). The results of this study showed that the changes in CI were relatively stable during subalpine training, whereas after subalpine training, CI increased significantly, and the athletes’ cardiac reserve was significantly enhanced.

### Stroke volume index

The beat-to-beat output test revealed that the athletes’ beat-to-beat output continued to decline after subalpine training. However, after leaving the subalpine environment, their beat-to-beat output returned to pre-subalpine levels, with no significant difference observed compared to pre-subalpine levels. In a separate study, Russian biathletes underwent three subalpine training sessions at an altitude of 1,100 m and three alternating plain transitions, with cardiovascular system tests performed in three phases during each subalpine and plain transition period. The results showed that from the first stage to the second stage for 57 days, the stroke output increased by 0.6% (P = 0.15), and from the second stage to the third stage for 52 days, the stroke output increased by 2.8% (P = 0.20), and there was no significant difference. The present study’s findings are consistent with the aforementioned study, as no significant difference was observed in output per beat after subalpine training compared to pre-subalpine levels. This outcome may be due to the athletes in this study undergoing continuous subalpine training without a recovery period, resulting in lower cardiovascular system stimulation compared to the alternating subalpine and plain training mode employed in the other study. The observed trend indicates that the athletes’ cardiac function was somewhat impacted upon entering subalpine training. This phenomenon may result from reduced cardiac function caused by hypoxia and low pressure, as systolic myocardial function is closely associated with the metabolic state of the myocardium itself, tissue structure, anterior and posterior load, and neurohumoral regulation (Faiss et al., 2015). Cardiac function is mainly influenced by the water in the body, and when first going to the subalpine, the human body experiences a decrease in blood volume and an increase in peripheral resistance, which in turn leads to a lack of effective circulating blood volume. Based on the findings of this study, It could be recommended that China’s top Paralympic biathletes undergoing subalpine training pay close attention to their cardiac output and heart rate indices while also focusing on output per beat. These athletes may enhance their heart function and output per beat after subalpine training by taking into account the specificity of physical function for disabled athletes in subalpine training (Yu et al., 2023). Due to the dry air and low humidity of the subalpine environment, it is crucial for athletes to continuously maintain good hydration status during subalpine training (Gore and Hahn, 2005). Studies have demonstrated that dehydration exceeding 1% of body weight impairs cognitive function, while dehydration exceeding 2% of body weight reduces aerobic capacity. It is important for athletes to monitor their hydration status closely and replenish fluids regularly during subalpine training (Mcdermott et al., 2017; Feng et al., 2022). Maintaining the proper hydration status of the body during subalpine training is an important prerequisite to ensure the quality of training and enhance athletic performance.

### Stroke volume variation

The beat-to-beat output variability test results indicated a significant increase in beat-to-beat output variability among the athletes after subalpine training, maintaining a certain level from weeks 2 to 6, and largely returning to pre-subalpine levels after leaving the subalpine environment, which was similar to the findings of the beat-to-beat output test. Some studies have suggested that a threshold of 10% for stroke volume variation (SVV) could be utilized to predict fluid response effectively, which can also serve as a threshold to distinguish between high and low SVV groups (Mehta et al., 2008). The present study’s results observed high SVV values, which may result from the low circulatory system volume during entry into subalpine training, low left heart preload, and the rising phase of the Frank-Starling curve. Fluctuations due to respiration may have a more significant effect on volume per beat, further contributing to the observed SVV values (de Wilde et al., 2009). SVV reflects changes in SV due to changes in intrathoracic pressure affecting the volume of blood returned to the heart, and increased blood viscosity leads to a further increase in capillary back resistance, which leads to a slowing of micro-bleeding in the body (Lewis et al., 2015). The above phenomena indicate that subalpine training increases the left ventricular preload of Paralympic biathletes and increases the body’s blood circulation volume and intra-airway and intrathoracic pressures. In the subalpine environment, the whole body systems of disabled athletes undergo different degrees of alterations, especially after high-intensity training, which is more complex than the functional changes in plains. Monitoring the variability index of output per beat might ensure an adequate hemodynamic capacity of the athletes and prevent hemodynamic disorders.

### Thoracic fluid content

The results of the pleural fluid level tests revealed that athletes’ fluid levels were significantly higher after their initial entry into the subalpine environment compared to pre-subalpine levels. However, these levels decreased significantly in the fourth week of subalpine training and remained stable until the end of the training period. The formation of inflammatory pleural effusions led to the development of plateau pulmonary edema upon initial entry into the plateau environment. An athlete with a pre-existing cold (ailment) before entering the plateau or sub-plateau was identified as a significant cause of inducing plateau pulmonary edema and pleural effusion. The present study’s findings suggest that athletes experienced a significant increase in thoracic fluid content upon their initial entry into the subalpine environment, which might be attributed to the stimulating effects of hypoxia and low pressure in the subalpine environment, coupled with training stimulation, which induced pulmonary vasoconstriction, elevated pulmonary arterial pressure, and increased pulmonary capillary pressure. These effects may lead to non-inflammatory leakage, resulting in interstitial and alveolar pulmonary edema. As the duration of subalpine training increased, the harmful effects of pulmonary edema gradually decreased, and the body developed a beneficial adaptation, leading to a reduction in the thoracic fluid content to pre-subalpine levels by the fourth week. It is recommended that during future subalpine training, Chinese Paralympic biathletes should be vigilant for the presence of inflammatory pleural effusion at the outset of the training, as well as any potential formation of plateau pulmonary edema (Schuler et al., 2007). Additionally, attention should be paid to the presence of pulmonary symptoms such as colds during the initial subalpine training period. and the training time and program should be promptly adjusted to prevent detrimental changes to the lungs from impacting the quality of training and athletic performance.

### Limitations

The present study has several limitations that must be addressed in future research. First, the study focused on the physiological performance of outstanding male Paralympic biathletes, and the results might not be generalizable to female athletes or athletes at different performance levels. Second, the study did not investigate the correlation between physiological changes and athletic performance, which could provide valuable insights into the effectiveness of subalpine training on sports performance. Third, the study did not specifically examine the characteristics of aerobic and anaerobic capacity, cardiac function, and hemodynamic changes in women’s subalpine training, which could reveal potential differences between male and female athletes. These could be addressed in future studies by conducting larger-scale investigations on both male and female athletes and examining the relationship between physiological changes and athletic performance.

## Conclusions

Eight weeks of 1,200 m subalpine training temporarily inhibits the aerobic capacity (significant decreases in VO_2_peak and VT in mid and late phases) of male paralympic biathletes, with only partial recovery post-altitude, suggesting that altitude training duration should be controlled and sufficient recovery time reserved.

Eight weeks of 1,200 m subalpine training reduces athletes’ anaerobic energy supply capacity and accelerates fatigue, with more significant impacts on sitting athletes (upper limb-dominated), indicating the need for personalized altitude training plans for athletes with different disability types.

Subalpine hypoxia alters athletes’ cardiac function and hemodynamics, which is synergistic with the decline in aerobic and anaerobic capacity, suggesting that cardiac function should be monitored during altitude training to guide training load adjustment.

## Data Availability

The raw data supporting the conclusions of this article will be made available by the authors, without undue reservation.
